# Poly[[[μ-3,3′-(dimethyl­silanedi­yl)dibenzoato][μ-1,1′-(1,4-phenyl­ene)di-1*H*-imidazole]­zinc] monohydrate]

**DOI:** 10.1107/S1600536812019642

**Published:** 2012-06-02

**Authors:** Guohui Huang, Yiling Bei

**Affiliations:** a Key Laboratory of Special Functional Aggregated Materials, Ministry of Education, School of Chemistry and Chemcail Engineering, Shandong University, Jinan 250100, People’s Republic of China

## Abstract

The asymmetric unit of the title compound, {[Zn(C_16_H_16_O_4_Si)(C_12_H_10_N_4_)]·H_2_O}_*n*_, consists of one Zn^II^ ion, two half 3,3′-(dimethyl­silanedi­yl)dibenzoate ligands and two half 1,1′-(1,4-phenyl­ene)di-1*H*-imidazole ligands. The Zn^II^ ion is four-coordinated by two O atoms from two carboxlate ligands, two N atoms from two imidazole ligands. Two Zn^II^ ions are bridged by two carboxyl­ate groups in chelating mode, generating a binuclear secondary building unit (SBU), which is further coordinated by two N atoms from two imidazole ligands in monodentate mode. Thus, the binuclear SBUs are further bridged by imidazole ligands in two different directions, giving rise to a chain. The water solvent mol­ecules are hydrogen bonded within the chain along the *c* axis.

## Related literature

For a similar presentation where the binuclear SBUs are further bridged by phenylenedicarboxylate ligands in different directions to give a three-dimensional porous framework containing three-dimensional channels, see: He *et al.* (2010 ▶).
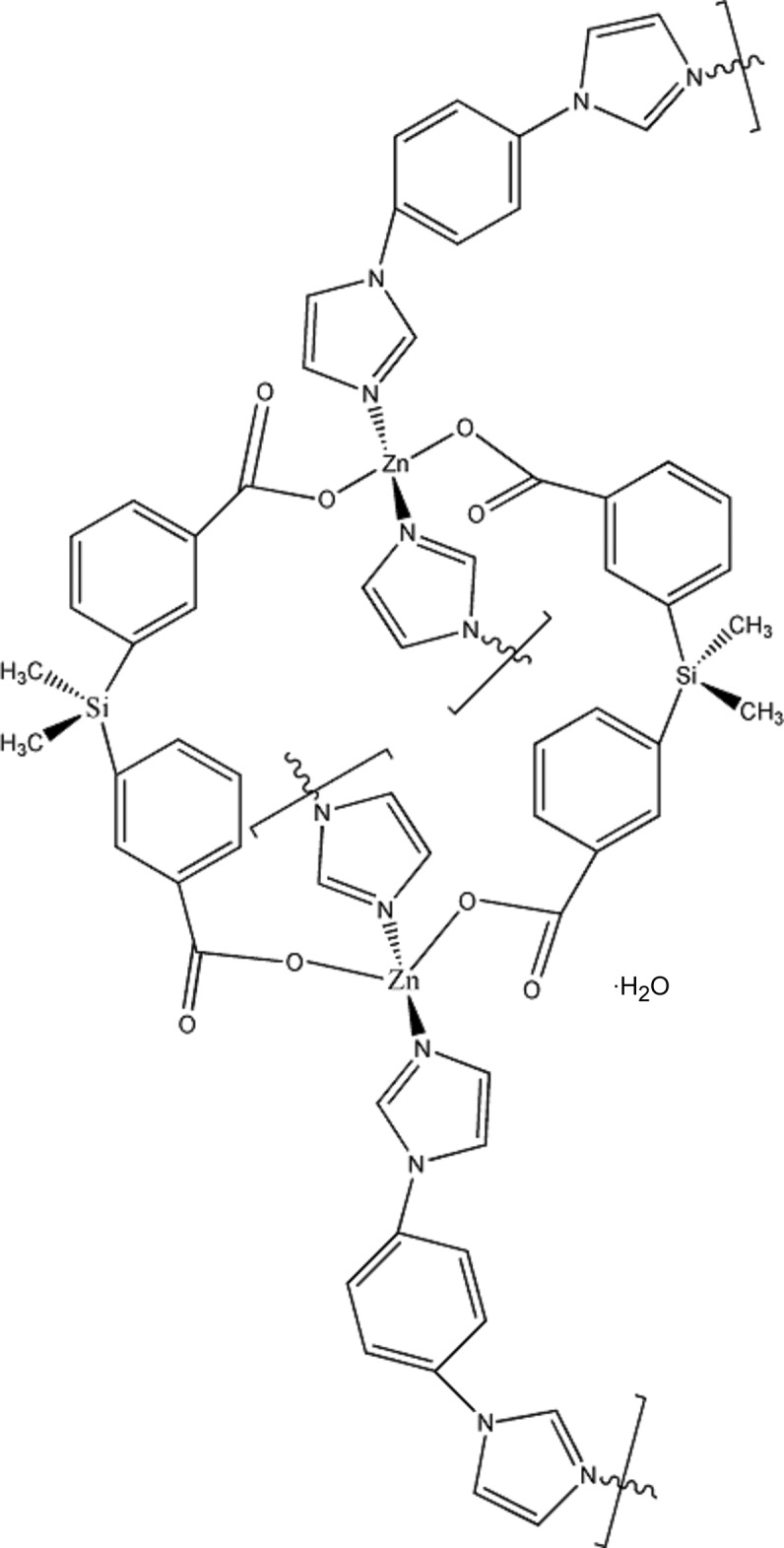


## Experimental

### 

#### Crystal data

[Zn(C_16_H_14_O_4_Si)(C_12_H_10_N_4_)]·H_2_O
*M* *_r_* = 591.99
Monoclinic, 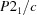

*a* = 9.4480 (11) Å
*b* = 23.959 (3) Å
*c* = 12.3083 (13) Å
β = 110.029 (2)°
*V* = 2617.7 (5) Å^3^
*Z* = 4
Mo *K*α radiation
μ = 1.03 mm^−1^
*T* = 298 K
0.15 × 0.10 × 0.05 mm

#### Data collection

Bruker APEXII CCD area-detector diffractometer
Absorption correction: multi-scan (*SADABS*; Sheldrick, 1996 ▶) *T*_min_ = 0.861, *T*_max_ = 0.950
15353 measured reflections
5881 independent reflections
3694 reflections with *I* > 2σ(*I*)
*R*_int_ = 0.054

#### Refinement

*R*[*F* ^2^ > 2σ(*F* ^2^)] = 0.053
*wR*(*F* ^2^) = 0.153
*S* = 1.02
5881 reflections
352 parameters
H-atom parameters constrained
Δρ_max_ = 0.69 e Å^−3^
Δρ_min_ = −0.74 e Å^−3^

### 

Data collection: *APEX2* (Bruker, 2005 ▶); cell refinement: *SAINT* (Bruker, 2005 ▶); data reduction: *SAINT*; program(s) used to solve structure: *SHELXS97* (Sheldrick, 2008 ▶); program(s) used to refine structure: *SHELXL97* (Sheldrick, 2008 ▶); molecular graphics: *SHELXTL* (Sheldrick, 2008 ▶); software used to prepare material for publication: *publCIF* (Westrip, 2010 ▶).

## Supplementary Material

Crystal structure: contains datablock(s) I, global. DOI: 10.1107/S1600536812019642/ds2187sup1.cif

Structure factors: contains datablock(s) test. DOI: 10.1107/S1600536812019642/ds2187Isup2.hkl

Additional supplementary materials:  crystallographic information; 3D view; checkCIF report

## Figures and Tables

**Table 1:** Hydrogen-bond geometry (Å, °)

*D*—H⋯*A*	*D*—H	H⋯*A*	*D*⋯*A*	*D*—H⋯*A*
O1*W*—H1*WB*⋯O2^i^	0.79	1.90	2.696 (8)	178
O1*W*—H1*WA*⋯O2	0.78	2.08	2.852 (8)	171

Symmetry code: (i) 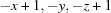.

**Fig. 1.**
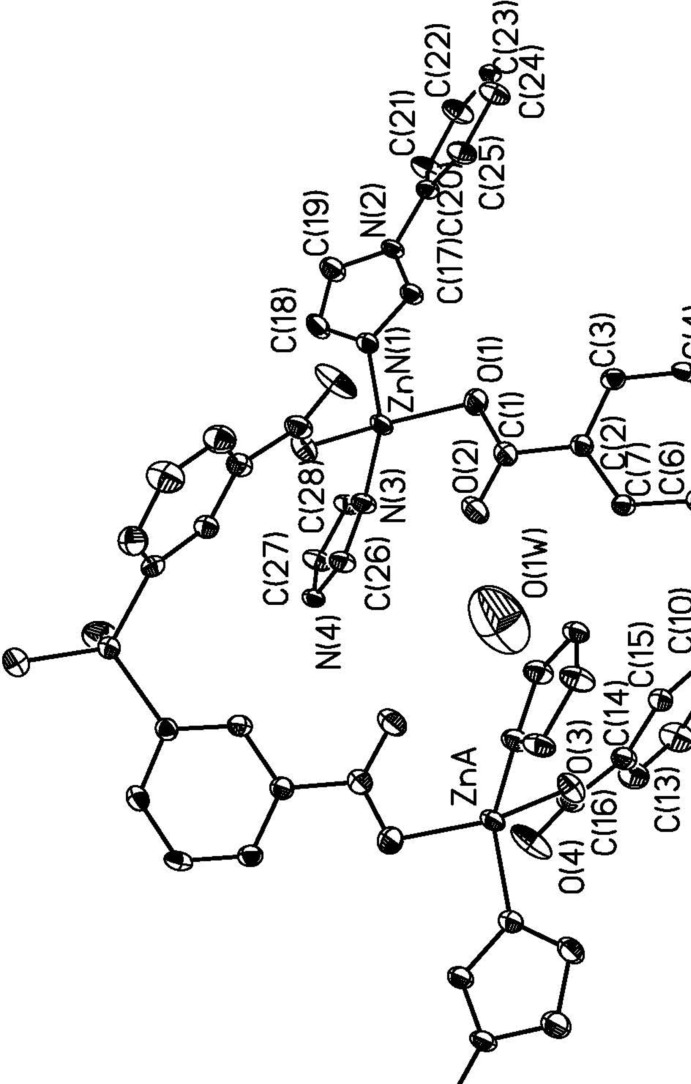
The asymmetric unit of the title compound.

**Fig. 2.**
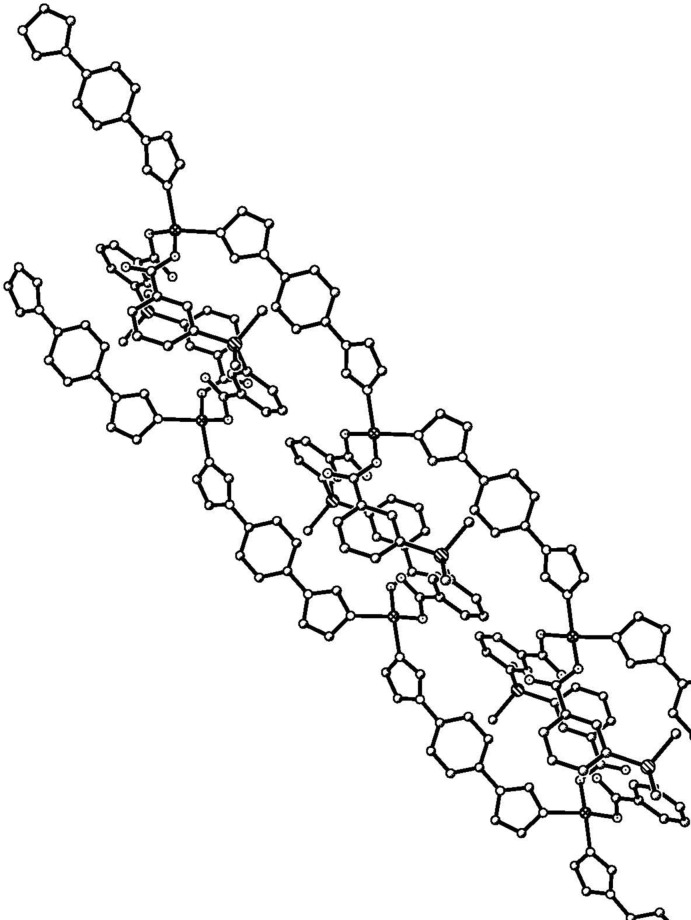
The one-dimensional chain of the title compound viewed by a axis.

**Fig. 3.**
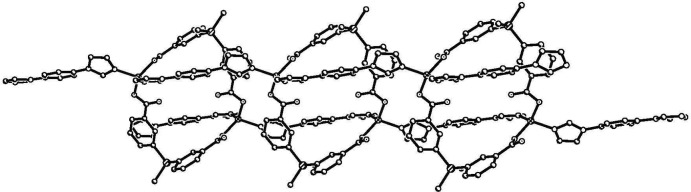
The one-dimensional chain of the title compound viewed by b axis.

**Fig. 4.**
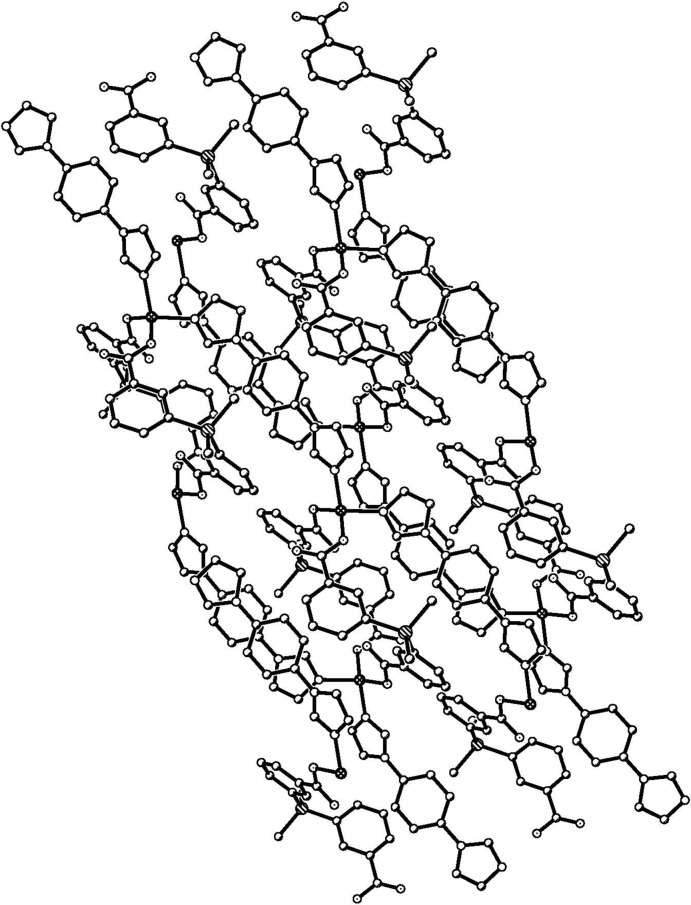
The packed structure of the title compound viewed by b axis.

## supplementary crystallographic information

### Experimental

4,4'-(Dimethylsilanediyl)dibenzoate (1 mg,0.0033 mmol), 1,4-bis(1-imidazolyl)benzene (10.8,0.0038 mmol) and Zn(NO_3_)_2_.6H_2_O (4 mg, 0.0159 mmol) were dissolved into 1 ml a mixed solution (DMF–EtOH–H2O = 1:1:1). The mixed solution was sealed into a Pyrex glass tube. The mixed solution was heated to 75 centigrade degree in 10 h, and kept the temperature at 75 centigrade degree for 72 h, and then was cooled down to room temperature in 10 h. colourless rod crystals were obtained. The crystals were filtered, washed with water and dried in air.

### Refinement

Water H atoms were located in a difference Fourier map and refined with distance restraints of O—H = 0.78 (2) Å and H···H = 1.37 (2) Å, and with Uiso(H) = 1.2Ueq(O). The carbon H-atoms were placed in calculated positions [C—H (aromatic) = 0.93 Å and C—H (methyl) = 0.96 Å] and were included in the refinement in the ridingmodel approximation, with Uiso(H) = 1.2Ueq(C)and 1.5Ueq(C) for methylene C—H.

### Crystal data

**Table:**

[Zn(C_16_H_14_O_4_Si)(C_12_H_10_N_4_)]·H_2_O	*F*(000) = 1224
*M**_r_* = 591.99	*D*_x_ = 1.502 Mg m^−^^3^
Monoclinic, *P*2_1_/*c*	Mo *K*α radiation, λ = 0.71073 Å
Hall symbol: -P 2ybc	Cell parameters from 2106 reflections
*a* = 9.4480 (11) Å	θ = 2.3–23.3°
*b* = 23.959 (3) Å	µ = 1.03 mm^−^^1^
*c* = 12.3083 (13) Å	*T* = 298 K
β = 110.029 (2)°	Rod, colourless
*V* = 2617.7 (5) Å^3^	0.15 × 0.10 × 0.05 mm
*Z* = 4	

### Data collection

**Table:**

Bruker APEXII CCD area-detector diffractometer	5881 independent reflections
Radiation source: fine-focus sealed tube	3694 reflections with *I* > 2σ(*I*)
Graphite monochromator	*R*_int_ = 0.054
phi and ω scans	θ_max_ = 27.5°, θ_min_ = 1.7°
Absorption correction: multi-scan (*SADABS*; Sheldrick, 1996)	*h* = −11→12
*T*_min_ = 0.861, *T*_max_ = 0.950	*k* = −29→30
15353 measured reflections	*l* = −15→9

### Refinement

**Table:**

Refinement on *F*^2^	Primary atom site location: structure-invariant direct methods
Least-squares matrix: full	Secondary atom site location: difference Fourier map
*R*[*F*^2^ > 2σ(*F*^2^)] = 0.053	Hydrogen site location: inferred from neighbouring sites
*wR*(*F*^2^) = 0.153	H-atom parameters constrained
*S* = 1.02	*w* = 1/[σ^2^(*F*_o_^2^) + (0.0685*P*)^2^ + 1.2685*P*] where *P* = (*F*_o_^2^ + 2*F*_c_^2^)/3
5881 reflections	(Δ/σ)_max_ < 0.001
352 parameters	Δρ_max_ = 0.69 e Å^−^^3^
0 restraints	Δρ_min_ = −0.74 e Å^−^^3^

### Special details

**Table:**

Geometry. All esds (except the esd in the dihedral angle between two l.s. planes) are estimated using the full covariance matrix. The cell esds are taken into account individually in the estimation of esds in distances, angles and torsion angles; correlations between esds in cell parameters are only used when they are defined by crystal symmetry. An approximate (isotropic) treatment of cell esds is used for estimating esds involving l.s. planes.
Refinement. Refinement of F^2^ against ALL reflections. The weighted R-factor wR and goodness of fit S are based on F^2^, conventional R-factors R are based on F, with F set to zero for negative F^2^. The threshold expression of F^2^ > 2sigma(F^2^) is used only for calculating R-factors(gt) etc. and is not relevant to the choice of reflections for refinement. R-factors based on F^2^ are statistically about twice as large as those based on F, and R- factors based on ALL data will be even larger.

### Fractional atomic coordinates and isotropic or equivalent isotropic displacement parameters (Å^2^)

**Table:**

	*x*	*y*	*z*	*U*_iso_*/*U*_eq_	
Zn	0.55041 (5)	−0.072654 (19)	0.85116 (4)	0.03338 (16)	
Si	0.77758 (13)	0.22634 (5)	0.67361 (9)	0.0332 (3)	
O1	0.6985 (3)	−0.01204 (12)	0.8930 (2)	0.0470 (8)	
O2	0.5877 (4)	0.01169 (15)	0.7116 (3)	0.0737 (12)	
O3	0.4631 (3)	0.13056 (12)	0.2612 (2)	0.0443 (7)	
O4	0.2206 (4)	0.13460 (18)	0.2361 (3)	0.0769 (12)	
N2	0.6888 (3)	−0.10157 (14)	1.2029 (2)	0.0316 (7)	
N1	0.5830 (4)	−0.09931 (13)	1.0146 (3)	0.0324 (7)	
N3	0.3262 (4)	−0.05579 (14)	0.7877 (3)	0.0355 (8)	
C1	0.6826 (5)	0.02014 (18)	0.8076 (4)	0.0406 (10)	
C2	0.7819 (4)	0.07052 (16)	0.8297 (3)	0.0309 (8)	
C3	0.9083 (4)	0.07470 (16)	0.9284 (3)	0.0322 (9)	
H3	0.9347	0.0450	0.9802	0.039*	
C4	0.9954 (4)	0.12246 (17)	0.9508 (3)	0.0359 (9)	
H4	1.0812	0.1247	1.0166	0.043*	
C5	0.9539 (4)	0.16695 (17)	0.8745 (3)	0.0369 (9)	
H5	1.0122	0.1992	0.8908	0.044*	
C6	0.8268 (4)	0.16490 (15)	0.7735 (3)	0.0285 (8)	
C7	0.7439 (4)	0.11537 (16)	0.7529 (3)	0.0306 (9)	
H7	0.6606	0.1122	0.6857	0.037*	
C8	0.9110 (5)	0.2296 (2)	0.5922 (4)	0.0617 (14)	
H8A	0.9026	0.1961	0.5476	0.093*	
H8B	1.0121	0.2332	0.6456	0.093*	
H8C	0.8871	0.2613	0.5413	0.093*	
C9	0.7933 (6)	0.29139 (19)	0.7606 (4)	0.0599 (14)	
H9A	0.7244	0.2895	0.8026	0.090*	
H9B	0.7692	0.3231	0.7100	0.090*	
H9C	0.8943	0.2950	0.8141	0.090*	
C10	0.5782 (4)	0.21887 (15)	0.5732 (3)	0.0328 (9)	
C11	0.4595 (5)	0.24408 (19)	0.5982 (4)	0.0447 (11)	
H11	0.4807	0.2645	0.6662	0.054*	
C13	0.2791 (5)	0.2091 (2)	0.4252 (4)	0.0489 (11)	
H13	0.1797	0.2062	0.3759	0.059*	
C14	0.3906 (4)	0.18279 (17)	0.3963 (3)	0.0352 (9)	
C15	0.5394 (4)	0.18807 (16)	0.4710 (3)	0.0323 (9)	
H15	0.6153	0.1704	0.4519	0.039*	
C16	0.3508 (5)	0.14646 (18)	0.2886 (3)	0.0400 (10)	
C18	0.5100 (5)	−0.13739 (19)	1.0587 (3)	0.0452 (11)	
H18	0.4282	−0.1588	1.0149	0.054*	
C19	0.5730 (5)	−0.13969 (19)	1.1743 (4)	0.0466 (11)	
H19	0.5444	−0.1623	1.2246	0.056*	
C17	0.6897 (4)	−0.07880 (16)	1.1052 (3)	0.0333 (9)	
H17	0.7576	−0.0515	1.1011	0.040*	
C20	0.7923 (4)	−0.08999 (16)	1.3165 (3)	0.0320 (9)	
C25	0.9402 (4)	−0.08007 (18)	1.3314 (3)	0.0393 (10)	
H25	0.9718	−0.0797	1.2676	0.047*	
C21	0.7454 (5)	−0.0894 (2)	1.4111 (4)	0.0515 (13)	
H21	0.6444	−0.0950	1.4012	0.062*	
C22	0.8485 (5)	−0.0804 (2)	1.5203 (3)	0.0482 (12)	
H22	0.8169	−0.0801	1.5840	0.058*	
C23	0.9971 (4)	−0.07189 (16)	1.5350 (3)	0.0308 (8)	
C24	1.0435 (5)	−0.07055 (19)	1.4409 (3)	0.0431 (11)	
H24	1.1437	−0.0633	1.4506	0.052*	
C27	0.0798 (5)	−0.06543 (18)	0.7533 (3)	0.0413 (10)	
H27	−0.0125	−0.0691	0.7640	0.050*	
C26	0.2541 (4)	−0.05871 (17)	0.6749 (3)	0.0375 (10)	
H26	0.3011	−0.0569	0.6198	0.045*	
C28	0.2151 (5)	−0.05982 (18)	0.8359 (3)	0.0401 (10)	
H28	0.2315	−0.0588	0.9148	0.048*	
N4	0.1048 (3)	−0.06458 (13)	0.6492 (2)	0.0309 (7)	
O1W	0.4293 (9)	−0.0453 (4)	0.5016 (6)	0.227 (4)	
H1WA	0.4808	−0.0308	0.5580	0.272*	
H1WB	0.4248	−0.0348	0.4393	0.272*	
C12	0.3113 (5)	0.2396 (2)	0.5253 (4)	0.0567 (13)	
H12	0.2346	0.2571	0.5438	0.068*	

### Atomic displacement parameters (Å^2^)

**Table:**

	*U*^11^	*U*^22^	*U*^33^	*U*^12^	*U*^13^	*U*^23^
Zn	0.0337 (3)	0.0394 (3)	0.0205 (2)	−0.0083 (2)	0.00091 (18)	−0.00123 (19)
Si	0.0376 (6)	0.0302 (6)	0.0275 (6)	−0.0039 (5)	0.0057 (5)	0.0010 (4)
O1	0.0557 (19)	0.0427 (18)	0.0385 (17)	−0.0169 (15)	0.0109 (14)	0.0076 (14)
O2	0.080 (3)	0.069 (2)	0.0442 (19)	−0.038 (2)	−0.0149 (18)	0.0108 (17)
O3	0.0414 (17)	0.0526 (19)	0.0376 (16)	−0.0095 (14)	0.0118 (14)	−0.0159 (14)
O4	0.0359 (19)	0.131 (4)	0.053 (2)	−0.016 (2)	0.0016 (16)	−0.033 (2)
N2	0.0305 (18)	0.0401 (19)	0.0190 (16)	−0.0025 (14)	0.0016 (13)	−0.0018 (13)
N1	0.0345 (18)	0.0347 (18)	0.0237 (16)	−0.0047 (15)	0.0045 (14)	0.0007 (14)
N3	0.0342 (19)	0.045 (2)	0.0221 (17)	−0.0042 (15)	0.0037 (14)	0.0011 (14)
C1	0.042 (3)	0.040 (2)	0.034 (2)	−0.0089 (19)	0.0054 (19)	0.0005 (19)
C2	0.031 (2)	0.033 (2)	0.0274 (19)	−0.0024 (17)	0.0079 (16)	0.0009 (16)
C3	0.036 (2)	0.035 (2)	0.0247 (19)	0.0021 (18)	0.0091 (16)	0.0038 (16)
C4	0.027 (2)	0.048 (3)	0.025 (2)	0.0016 (18)	−0.0008 (16)	0.0034 (18)
C5	0.036 (2)	0.035 (2)	0.036 (2)	−0.0087 (18)	0.0074 (18)	−0.0048 (18)
C6	0.030 (2)	0.029 (2)	0.0262 (19)	0.0001 (16)	0.0088 (16)	−0.0032 (15)
C7	0.028 (2)	0.037 (2)	0.0257 (19)	−0.0011 (17)	0.0069 (16)	0.0021 (16)
C8	0.058 (3)	0.081 (4)	0.047 (3)	−0.010 (3)	0.019 (2)	0.014 (3)
C9	0.068 (3)	0.041 (3)	0.051 (3)	−0.003 (2)	−0.004 (2)	−0.007 (2)
C10	0.041 (2)	0.027 (2)	0.026 (2)	0.0022 (17)	0.0070 (17)	0.0051 (16)
C11	0.050 (3)	0.046 (3)	0.040 (2)	0.006 (2)	0.017 (2)	−0.005 (2)
C13	0.035 (2)	0.063 (3)	0.045 (3)	0.004 (2)	0.008 (2)	−0.002 (2)
C14	0.034 (2)	0.040 (2)	0.030 (2)	−0.0005 (18)	0.0079 (17)	0.0005 (17)
C15	0.032 (2)	0.035 (2)	0.029 (2)	0.0008 (17)	0.0090 (17)	0.0001 (16)
C16	0.039 (3)	0.051 (3)	0.026 (2)	−0.006 (2)	0.0060 (19)	0.0020 (18)
C18	0.045 (3)	0.053 (3)	0.030 (2)	−0.022 (2)	0.0032 (19)	−0.0019 (19)
C19	0.047 (3)	0.056 (3)	0.032 (2)	−0.017 (2)	0.008 (2)	0.003 (2)
C17	0.032 (2)	0.041 (2)	0.0226 (19)	−0.0068 (17)	0.0039 (16)	0.0017 (17)
C20	0.032 (2)	0.037 (2)	0.0217 (19)	0.0009 (17)	0.0030 (16)	0.0003 (16)
C25	0.034 (2)	0.059 (3)	0.025 (2)	0.005 (2)	0.0097 (17)	−0.0044 (18)
C21	0.032 (2)	0.091 (4)	0.029 (2)	−0.012 (2)	0.0069 (19)	0.002 (2)
C22	0.039 (3)	0.083 (4)	0.021 (2)	−0.011 (2)	0.0096 (18)	0.001 (2)
C23	0.031 (2)	0.034 (2)	0.0217 (18)	0.0015 (17)	0.0008 (15)	0.0006 (16)
C24	0.030 (2)	0.068 (3)	0.028 (2)	0.000 (2)	0.0059 (17)	−0.007 (2)
C27	0.035 (2)	0.063 (3)	0.024 (2)	0.003 (2)	0.0081 (17)	−0.0039 (19)
C26	0.036 (2)	0.050 (3)	0.025 (2)	−0.0019 (19)	0.0077 (17)	0.0018 (17)
C28	0.043 (3)	0.052 (3)	0.020 (2)	0.002 (2)	0.0039 (18)	−0.0032 (17)
N4	0.0306 (18)	0.0365 (19)	0.0209 (15)	0.0005 (14)	0.0029 (13)	−0.0005 (13)
O1W	0.238 (9)	0.309 (11)	0.153 (6)	−0.097 (8)	0.092 (6)	−0.052 (7)
C12	0.042 (3)	0.075 (4)	0.054 (3)	0.017 (3)	0.017 (2)	−0.010 (3)

### Geometric parameters (Å, º)

**Table:**

Zn—O3^i^	1.932 (3)	C9—H9C	0.9600
Zn—O1	1.959 (3)	C10—C15	1.395 (5)
Zn—N1	2.031 (3)	C10—C11	1.398 (6)
Zn—N3	2.032 (3)	C11—C12	1.384 (6)
Si—C8	1.862 (5)	C11—H11	0.9300
Si—C9	1.868 (5)	C13—C12	1.374 (6)
Si—C6	1.872 (4)	C13—C14	1.375 (6)
Si—C10	1.875 (4)	C13—H13	0.9300
O1—C1	1.271 (5)	C14—C15	1.397 (5)
O2—C1	1.231 (5)	C14—C16	1.522 (6)
O3—C16	1.276 (5)	C15—H15	0.9300
O3—Zn^i^	1.932 (3)	C18—C19	1.342 (5)
O4—C16	1.212 (5)	C18—H18	0.9300
N2—C17	1.324 (5)	C19—H19	0.9300
N2—C19	1.375 (5)	C17—H17	0.9300
N2—C20	1.432 (4)	C20—C25	1.367 (5)
N1—C17	1.316 (5)	C20—C21	1.380 (5)
N1—C18	1.363 (5)	C25—C24	1.386 (5)
N3—C26	1.322 (5)	C25—H25	0.9300
N3—C28	1.375 (5)	C21—C22	1.380 (6)
C1—C2	1.495 (5)	C21—H21	0.9300
C2—C3	1.385 (5)	C22—C23	1.368 (6)
C2—C7	1.394 (5)	C22—H22	0.9300
C3—C4	1.381 (5)	C23—C24	1.372 (5)
C3—H3	0.9300	C23—N4^ii^	1.436 (4)
C4—C5	1.386 (5)	C24—H24	0.9300
C4—H4	0.9300	C27—C28	1.339 (5)
C5—C6	1.403 (5)	C27—N4	1.380 (5)
C5—H5	0.9300	C27—H27	0.9300
C6—C7	1.396 (5)	C26—N4	1.343 (5)
C7—H7	0.9300	C26—H26	0.9300
C8—H8A	0.9600	C28—H28	0.9300
C8—H8B	0.9600	N4—C23^iii^	1.436 (4)
C8—H8C	0.9600	O1W—H1WA	0.7791
C9—H9A	0.9600	O1W—H1WB	0.7946
C9—H9B	0.9600	C12—H12	0.9300
			
O3^i^—Zn—O1	125.92 (13)	C12—C11—C10	122.2 (4)
O3^i^—Zn—N1	115.69 (13)	C12—C11—H11	118.9
O1—Zn—N1	96.91 (12)	C10—C11—H11	118.9
O3^i^—Zn—N3	93.61 (13)	C12—C13—C14	121.5 (4)
O1—Zn—N3	120.70 (13)	C12—C13—H13	119.3
N1—Zn—N3	103.17 (13)	C14—C13—H13	119.3
C8—Si—C9	109.8 (3)	C13—C14—C15	118.4 (4)
C8—Si—C6	109.0 (2)	C13—C14—C16	120.4 (4)
C9—Si—C6	109.04 (19)	C15—C14—C16	121.2 (4)
C8—Si—C10	111.2 (2)	C10—C15—C14	122.4 (4)
C9—Si—C10	108.8 (2)	C10—C15—H15	118.8
C6—Si—C10	108.99 (17)	C14—C15—H15	118.8
C1—O1—Zn	111.1 (3)	O4—C16—O3	125.0 (4)
C16—O3—Zn^i^	123.1 (3)	O4—C16—C14	120.1 (4)
C17—N2—C19	107.0 (3)	O3—C16—C14	114.9 (4)
C17—N2—C20	126.3 (3)	C19—C18—N1	110.4 (4)
C19—N2—C20	126.6 (3)	C19—C18—H18	124.8
C17—N1—C18	104.9 (3)	N1—C18—H18	124.8
C17—N1—Zn	122.3 (3)	C18—C19—N2	105.6 (4)
C18—N1—Zn	132.8 (3)	C18—C19—H19	127.2
C26—N3—C28	104.7 (3)	N2—C19—H19	127.2
C26—N3—Zn	119.2 (3)	N1—C17—N2	112.0 (3)
C28—N3—Zn	132.0 (3)	N1—C17—H17	124.0
O2—C1—O1	122.4 (4)	N2—C17—H17	124.0
O2—C1—C2	120.8 (4)	C25—C20—C21	119.6 (4)
O1—C1—C2	116.7 (3)	C25—C20—N2	119.3 (3)
C3—C2—C7	118.9 (3)	C21—C20—N2	121.1 (4)
C3—C2—C1	121.1 (3)	C20—C25—C24	120.4 (4)
C7—C2—C1	120.0 (3)	C20—C25—H25	119.8
C4—C3—C2	120.7 (4)	C24—C25—H25	119.8
C4—C3—H3	119.6	C22—C21—C20	120.0 (4)
C2—C3—H3	119.6	C22—C21—H21	120.0
C3—C4—C5	119.4 (4)	C20—C21—H21	120.0
C3—C4—H4	120.3	C23—C22—C21	120.1 (4)
C5—C4—H4	120.3	C23—C22—H22	119.9
C4—C5—C6	122.1 (4)	C21—C22—H22	119.9
C4—C5—H5	118.9	C24—C23—C22	120.1 (3)
C6—C5—H5	118.9	C24—C23—N4^ii^	120.0 (3)
C7—C6—C5	116.6 (3)	C22—C23—N4^ii^	119.9 (3)
C7—C6—Si	123.5 (3)	C23—C24—C25	119.7 (4)
C5—C6—Si	120.0 (3)	C23—C24—H24	120.1
C2—C7—C6	122.3 (3)	C25—C24—H24	120.1
C2—C7—H7	118.9	C28—C27—N4	106.3 (4)
C6—C7—H7	118.9	C28—C27—H27	126.8
Si—C8—H8A	109.5	N4—C27—H27	126.8
Si—C8—H8B	109.5	N3—C26—N4	112.0 (4)
H8A—C8—H8B	109.5	N3—C26—H26	124.0
Si—C8—H8C	109.5	N4—C26—H26	124.0
H8A—C8—H8C	109.5	C27—C28—N3	110.5 (4)
H8B—C8—H8C	109.5	C27—C28—H28	124.7
Si—C9—H9A	109.5	N3—C28—H28	124.7
Si—C9—H9B	109.5	C26—N4—C27	106.4 (3)
H9A—C9—H9B	109.5	C26—N4—C23^iii^	125.3 (3)
Si—C9—H9C	109.5	C27—N4—C23^iii^	128.1 (3)
H9A—C9—H9C	109.5	H1WA—O1W—H1WB	122.0
H9B—C9—H9C	109.5	C13—C12—C11	119.2 (4)
C15—C10—C11	116.4 (4)	C13—C12—H12	120.4
C15—C10—Si	122.7 (3)	C11—C12—H12	120.4
C11—C10—Si	121.0 (3)		
			
O3^i^—Zn—O1—C1	64.2 (3)	C12—C13—C14—C16	176.4 (4)
N1—Zn—O1—C1	−166.9 (3)	C11—C10—C15—C14	0.6 (6)
N3—Zn—O1—C1	−57.1 (3)	Si—C10—C15—C14	−179.8 (3)
O3^i^—Zn—N1—C17	129.7 (3)	C13—C14—C15—C10	0.1 (6)
O1—Zn—N1—C17	−5.8 (3)	C16—C14—C15—C10	−176.8 (4)
N3—Zn—N1—C17	−129.6 (3)	Zn^i^—O3—C16—O4	−12.7 (6)
O3^i^—Zn—N1—C18	−51.9 (4)	Zn^i^—O3—C16—C14	167.1 (3)
O1—Zn—N1—C18	172.6 (4)	C13—C14—C16—O4	−9.6 (7)
N3—Zn—N1—C18	48.7 (4)	C15—C14—C16—O4	167.2 (4)
O3^i^—Zn—N3—C26	−36.1 (3)	C13—C14—C16—O3	170.6 (4)
O1—Zn—N3—C26	100.1 (3)	C15—C14—C16—O3	−12.6 (6)
N1—Zn—N3—C26	−153.5 (3)	C17—N1—C18—C19	−0.3 (5)
O3^i^—Zn—N3—C28	117.6 (4)	Zn—N1—C18—C19	−178.9 (3)
O1—Zn—N3—C28	−106.3 (4)	N1—C18—C19—N2	0.2 (5)
N1—Zn—N3—C28	0.2 (4)	C17—N2—C19—C18	−0.1 (5)
Zn—O1—C1—O2	−2.0 (6)	C20—N2—C19—C18	−178.1 (4)
Zn—O1—C1—C2	176.0 (3)	C18—N1—C17—N2	0.2 (5)
O2—C1—C2—C3	−166.5 (4)	Zn—N1—C17—N2	179.0 (3)
O1—C1—C2—C3	15.5 (6)	C19—N2—C17—N1	−0.1 (5)
O2—C1—C2—C7	16.5 (6)	C20—N2—C17—N1	177.9 (4)
O1—C1—C2—C7	−161.5 (4)	C17—N2—C20—C25	−36.5 (6)
C7—C2—C3—C4	0.1 (6)	C19—N2—C20—C25	141.1 (4)
C1—C2—C3—C4	−177.0 (4)	C17—N2—C20—C21	144.3 (4)
C2—C3—C4—C5	1.2 (6)	C19—N2—C20—C21	−38.0 (6)
C3—C4—C5—C6	−1.0 (6)	C21—C20—C25—C24	1.3 (7)
C4—C5—C6—C7	−0.5 (6)	N2—C20—C25—C24	−177.9 (4)
C4—C5—C6—Si	−179.7 (3)	C25—C20—C21—C22	−1.9 (7)
C8—Si—C6—C7	−104.7 (3)	N2—C20—C21—C22	177.3 (4)
C9—Si—C6—C7	135.5 (3)	C20—C21—C22—C23	0.2 (7)
C10—Si—C6—C7	16.9 (4)	C21—C22—C23—C24	2.0 (7)
C8—Si—C6—C5	74.5 (4)	C21—C22—C23—N4^ii^	−177.7 (4)
C9—Si—C6—C5	−45.3 (4)	C22—C23—C24—C25	−2.6 (6)
C10—Si—C6—C5	−164.0 (3)	N4^ii^—C23—C24—C25	177.2 (4)
C3—C2—C7—C6	−1.6 (6)	C20—C25—C24—C23	0.9 (7)
C1—C2—C7—C6	175.5 (4)	C28—N3—C26—N4	−0.3 (5)
C5—C6—C7—C2	1.8 (5)	Zn—N3—C26—N4	159.8 (3)
Si—C6—C7—C2	−179.0 (3)	N4—C27—C28—N3	−0.4 (5)
C8—Si—C10—C15	35.2 (4)	C26—N3—C28—C27	0.4 (5)
C9—Si—C10—C15	156.2 (3)	Zn—N3—C28—C27	−156.0 (3)
C6—Si—C10—C15	−85.0 (3)	N3—C26—N4—C27	0.1 (5)
C8—Si—C10—C11	−145.2 (4)	N3—C26—N4—C23^iii^	−176.5 (3)
C9—Si—C10—C11	−24.2 (4)	C28—C27—N4—C26	0.2 (5)
C6—Si—C10—C11	94.6 (4)	C28—C27—N4—C23^iii^	176.7 (4)
C15—C10—C11—C12	−0.9 (6)	C14—C13—C12—C11	0.3 (8)
Si—C10—C11—C12	179.5 (4)	C10—C11—C12—C13	0.5 (7)
C12—C13—C14—C15	−0.5 (7)		

Symmetry codes: (i) −*x*+1, −*y*, −*z*+1; (ii) *x*+1, *y*, *z*+1; (iii) *x*−1, *y*, *z*−1.

### Hydrogen-bond geometry (Å, º)

**Table:**

*D*—H···*A*	*D*—H	H···*A*	*D*···*A*	*D*—H···*A*
O1*W*—H1*WB*···O2^i^	0.79	1.90	2.696 (8)	178
O1*W*—H1*WA*···O2	0.78	2.08	2.852 (8)	171

Symmetry code: (i) −*x*+1, −*y*, −*z*+1.
